# Enriched metabolites that potentially promote age-associated diseases in subjects with an elderly-type gut microbiota

**DOI:** 10.1080/19490976.2020.1865705

**Published:** 2021-01-11

**Authors:** Shin Yoshimoto, Eri Mitsuyama, Keisuke Yoshida, Toshitaka Odamaki, Jin-zhong Xiao

**Affiliations:** Next Generation Science Institute, Morinaga Milk Industry Co., Ltd., Kanagawa, Japan

**Keywords:** Word: elderly-type fecal metabolite, age-related gut microbiota cluster, age-associated disease, intestinal and systemic homeostasis, TMA-producing bacteria

## Abstract

We previously investigated the gut microbiota of 453 healthy Japanese subjects aged 0 to 104 years and found that the composition of the gut microbiota could be classified into some age-related clusters. In this study, we compared fecal metabolites between age-matched and age-mismatched elderly subjects to examine the roles of the gut microbiota in the health of the elderly. Fecal metabolites in 16 elderly subjects who fell into an age-matched cluster (elderly-type gut microbiota group, E-GM) and another 16 elderly subjects who fell into an age-mismatched cluster (adult-type gut microbiota group, A-GM) were measured by CE-TOF-MS. A total of eight metabolites were significantly different between the groups: cholic acid and taurocholic acid were enriched in the A-GM group, whereas choline, trimethylamine (TMA), N8-acetylspermidine, propionic acid, 2-hydroxy-4-methylvaleric acid, and 5-methylcytosine were enriched in the E-GM group. Some metabolites (choline, TMA, N8-acetylspermidine) elevated in the E-GM group were metabolites or precursors reported as risk factors for age-associated diseases such as arteriosclerosis and colorectal cancer. The abundance of some species belongs to *Proteobacteria*, which were known as TMA-producing bacteria, was increased in the E-GM group and correlated with fecal TMA levels. *In vitro* assays showed that these elderly-type fecal metabolites suppressed the expression of genes related to tight junctions in normal colonic epithelial cells and induced the expression of inflammatory cytokines in colon cancer cells. These findings suggest that metabolites produced by the aged gut microbiota could contribute to intestinal and systemic homeostasis and could be targeted for preventing aging-associated diseases.

## Introduction

Commensal microbes and their multicellular eukaryotic hosts constitute a highly integrated system, and a growing number of studies suggest that the intestinal microbiota plays an important role in host health and disease. In recent decades, it has become clear that the gut microbiota changes with aging in heathy subjects. The microbial composition in the gut of newborns is dramatically shaped by diet and varies depending on whether the infant is fed maternal milk or formula.^1,2^ During adult life, in the absence of external disturbances, the gut microbiota is relatively stable, but an altered composition has been repeatedly reported in elderly subjects.^2^ We previously found age-dependent changes in the gut microbiota of healthy Japanese subjects aged 0 to 104 years and demonstrated a decrease in obligate anaerobes such as *Bifidobacterium* but an increase in facultative anaerobes such as *Escherichia coli* in the elderly.^3^

Concomitant with microbiota changes, it is well known that homeostasis between proinflammatory and regulatory responses is lost in the elderly, which results in a state of chronic low-grade systemic inflammation.^4^ Age-dependent chronic low-grade inflammation, which is called inflammaging, is a risk factor for many age-associated chronic diseases, such as cardiovascular diseases (CVDs), chronic kidney disease, diabetes mellitus, sarcopenia, depression, dementia, and cancer.^5^ Recently, the aged gut microbiota and intestinal permeability have been noted as being causes of inflammaging. Inflammaging can be exacerbated in germ-free mice by gut microbiota transfers from aged donor mice.^6^ Moreover, lipopolysaccharide (LPS) from the gut microbiota can accelerate inflammaging,^5^ and mice lacking Toll-like receptor 4 (TLR4) are protected from age-dependent inflammation,^7^ showing a direct causal relation between age-specific microbial communities and host age-associated phenotypes based on chronic inflammation. However, other than the effect of LPS, the effects of the aged gut microbiota on age-dependent chronic inflammation are unknown.

In this study, we compared fecal metabolites between healthy elderly subjects who had an age-matched or age-mismatched gut microbiota to identify characteristic metabolites derived from the age-matched gut microbiota. In addition, the possible function of these metabolites in the gut was evaluated using human colonic epithelial cells.

## Results

### Subject selection based on gut microbiota composition

**Figure 1.**
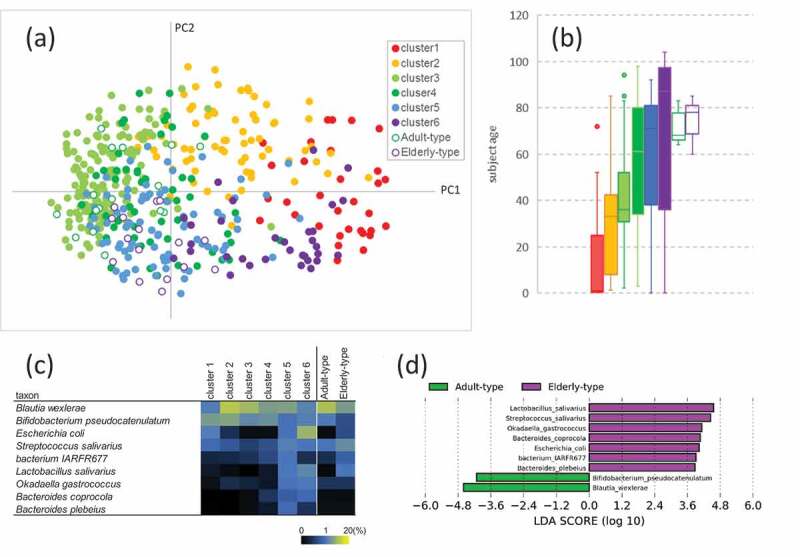
Fecal microbiota composition. (a) Principal component analysis (PCoA) based on Jensen-Shannon divergence. (b) Box plot of subject age in each cluster shown in Figure 1a. Lines in the boxes in the graph represent the 25th, median, and 75th percentiles; whiskers extend to the maximum and minimum values within 1.5x the interquartile range; and dots indicate outliers. (c) Average abundance of bacterial taxa that were significantly different between the adult- and elderly-type groups (d) Histogram of the LDA scores (log10) computed for features with differential abundance in adult- and elderly-type groups

We previously investigated the gut microbiota composition of 453 healthy Japanese subjects aged 0 to 104 years.^8^ Based on the obtained species-level gut microbiota composition of these subjects, we performed principal coordinate analysis (PCoA) and classified the subjects into six clusters (Figure 1a). Subjects were predominantly clustered by age. The median (interquartile range) age of each cluster was 0.9 (0.33–11), 33.0 (8–41), 36.0 (31–51.8), 61.0 (34.8–79.8), 71.0 (38–81) and 87.0 (62–95.8) in clusters 1, 2, 3, 4, 5 and 6, respectively (Figure 1b). However, we found that some subjects fell into age-mismatched gut microbiota clusters. We focused on some elderly subjects who fell into age-mismatched gut microbiota clusters (2, 3, and 4, mainly composed of adult subjects). To understand the characteristic fecal metabolites derived from the aged gut microbiota, we compared the fecal metabolite profiles of 16 elderly subjects with an age-matched gut microbiota (in cluster 6, referred to as the elderly-type gut microbiota, E-GM group) and 16 elderly subjects with an age-mismatched gut microbiota (in clusters 3 and 4, referred as adult-type gut microbiota, A-GM group) (Figure 1a). No significant difference in the actual age was observed between the E-GM group (75.1 ± 7.6 years) and the A-GM group (71.6 ± 6.5 years) (Figure 1b). In contrast, the abundances of the predominant species *Blautia wexlerae* and *Bifidobacterium pseudocatenulatum* were significantly higher in the A-GM group, whereas a higher abundance of facultative anaerobic bacteria, such as *Escherichia coli, Streptococcus salivarius*, and *Lactobacillus salivarius*, was observed in the E-GM group (Figure 1c,d; Supplementary Fig. 1 and 2). In total, nine species were significantly different between the two groups (Figure 1c,d).

### Metabolomic analysis

**Figure 2.**
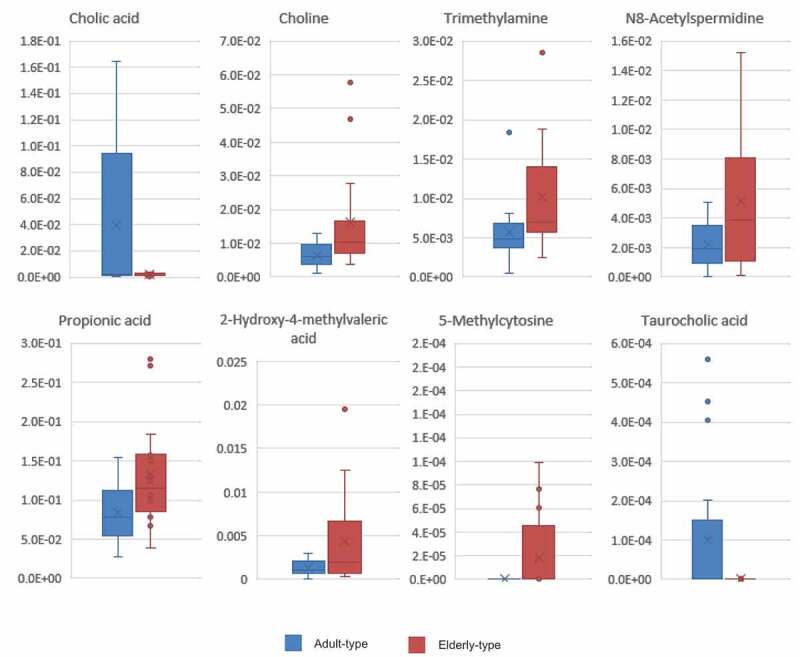
Eight fecal metabolites were significantly different between the adult- and elderly-type gut microbiota. Relative peak areas of indicated metabolites in the adult- and elderly-type gut microbiota groups. Lines in the boxes in the graph represent the 25th, median, and 75th percentiles; whiskers extend to the maximum and minimum values within 1.5x the interquartile range; and dots indicate outliers (Welch’s t-test <0.05)

Fecal metabolomic analysis by capillary electrophoresis time-of-flight mass spectrometry (CE-TOF-MS) showed a total of 351 candidate compound peaks. Of these compounds, eight metabolites were significantly different between the two groups (Figure 2). The level of cholic acid, a primary bile acid, was 1.5 times higher in the A-GM group than in the E-GM group, and taurocholic acid, which is a taurine-conjugated form of cholic acid, was detected only in four samples with high relative cholic acid levels in the A-GM group. The level of trimethylamine (TMA), the precursor of trimethylamine N-oxide (TMAO), which has been attracting attention as a risk factor for cardiovascular disorders, was 1.4 times higher in the E-GM group than in the A-GM group. Similarly, the level of choline, the precursor of TMA, was 1.7 times higher in the E-GM group than in the A-GM group. The levels of propionic acid and 2-hydroxy-4-methylvaleric acid, related to short-chain fatty acids, were elevated by 1.4 times and 1.7 times, respectively, in the E-GM group. N8-acetylspermidine, a polyamine metabolite, was approximately twice as abundant in the E-GM group compared with the A-GM group. 5-methylcytosine was detected in only four samples in the E-GM group.

### Relationship between metabolites and the gut microbiota

Since all of these metabolites are metabolites of or will be metabolized by intestinal bacteria, the correlation of the relative levels of the other six metabolites with the relative abundance of the intestinal bacterial species was investigated with the exception of taurocholic acid and 5-methylcytosine, which were produced at low levels. A significantly negative correlation with cholic acid was observed in *Akkermansia muciniphila*. Moreover, *Klebsiella oxytoca*, which was abundant in the E-GM group (A-GM vs E-GM; 0.00 ± 0.012% vs 0.02 ± 0.059%, *p* < 0.05), had a significant positive correlation with fecal TMA production.

### Effects on normal colonic epithelial cells and colon cancer cells

To examine the effect of each metabolite on the host, the concentration of each metabolite in feces was assessed. Since the levels of taurocholic acid and 5-methylcytosine were undetectable and standard substances are not available for 2-hydroxy-4-methylvaleric acid, the concentrations of the remaining five metabolites were measured using three samples out of 32 samples. It was found that the actual measured values for each substance showed a tendency similar to that observed in the CE-TOF-MS analysis. Table 1 shows the fecal metabolite concentrations of choline, TMA, N8-acetylspermidine, propionic acid, and cholic acid in both groups calculated from the results of CE-TOF-MS analysis of each sample with reference to the measured values of the three samples.

**Table 1.** Concentration of each metabolite in feces

	choline	trimethylamine	N^8^-acetylspermidine	cholic acid	propionic acid
A-GM group (nmol/g)	54.02	75.92	7.40	37.51	11170.28
E-GM group (nmol/g)	91.49	109.87	11.56	24.77	16368.91

Using 3 samples, each fecal metabolite concentration was measured by CE-TOF-MS using the standard substances. The values for the remaining samples were calculated based on the relative peak area, and the median of both groups is shown.

**Figure 3.**
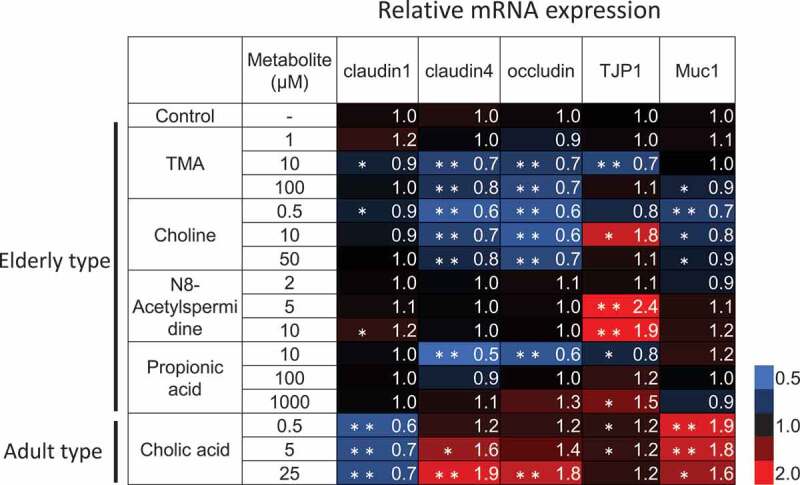
Downregulation of epithelial barrier-related gene expression by elderly-type gut metabolites in normal colonic epithelial cells. HCoEpiCs were cultured with cell culture medium containing the indicated metabolite or distilled water for 3 days. These cells were then subjected to RT-qPCR for the indicated genes. Data represent the mean of three independent experiments, each performed in triplicate, and are presented relative to the control. Asterisks indicate the statistical significance of each concentration of metabolite versus distilled water (* P<0.05, ** P<0.01; Tukey’s test)

We then performed an *in vitro* assay using primary human normal colonic epithelial cells (HCoEpiCs) to verify the possible functions of these metabolites in intestinal homeostasis. Since the mucosal layer acts as a barrier in the intestine, each metabolite was added to the colonic epithelial cells at a lower concentration than that detected in the fecal samples. Among the E-GM group-enriched metabolites, we found that TMA, choline, and propionic acid suppressed the expression of tight junction-related genes, especially claudin-4 and occludin. On the other hand, cholic acid, enriched in the A-GM group, tended to increase the expression of these genes (Figure 3). These results indicate that the accumulation of elderly-type intestinal metabolites might increase intestinal permeability in healthy subjects.

**Figure 4.**
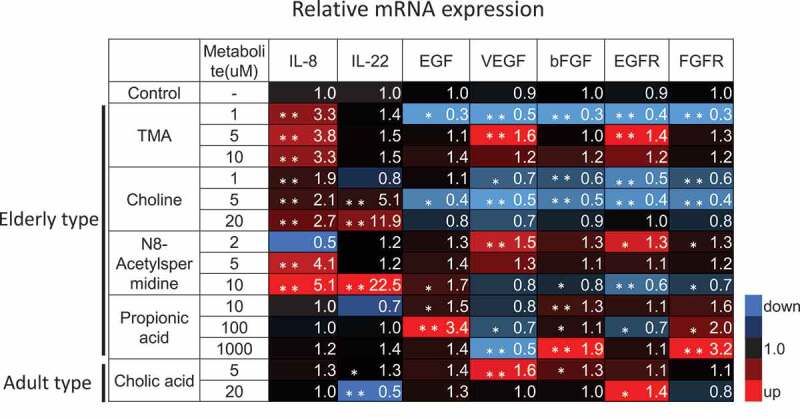
Induction of cytokines and growth factors expression by the elderly-type gut metabolites in colon cancer cells. HCT116 cells were cultured with cell culture medium containing the indicated metabolite or distilled water for 3 days. These cells were then subjected to RT-qPCR for indicated genes. Data represent the mean (± standard deviation, SD) of three independent experiments, each performed in triplicate, and are presented relative to the control. Error bars indicate SDs. Asterisks indicate the statistical significance of each concentration of metabolite versus distilled water (* P<0.05, ** P<0.01; Tukey’s test)

**Figure 5.**
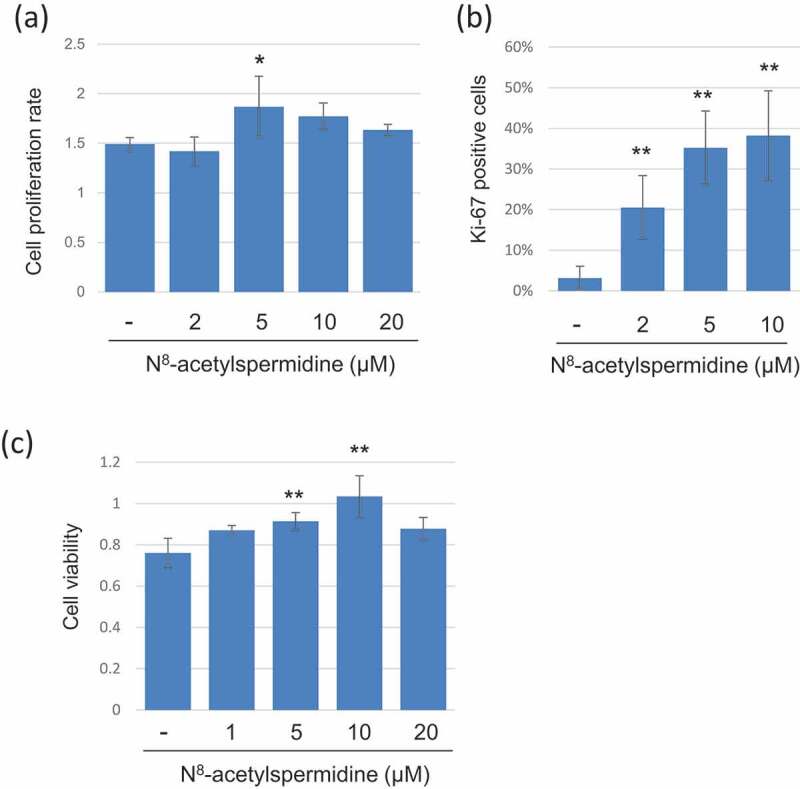
Induction of cell growth and chemoresistance to colon cancer cells by N8-acetylspermidine. (a) HCT116 cells were cultured with FBS-free culture medium containing N8-acetylspermidine, and then, cell viability was measured using CCK8 kit at 24 h and 72 h. The cell proliferation rate was calculated as the cell viability at 72h/cell viability at 24 h. (b) HCT116 cells were cultured with FBS-free culture medium containing N8-acetylspermidine for 72h, and the cells were immunocytochemistry stained with proliferation marker protein Ki-67 and DAPI. The percentage of Ki-67 positive cells was counted from randomly taken 10 images. (c) HCT116 cells were cultured with culture medium containing 20 µM oxaliplatin and N8-acetylspermidine. After 48 h, the cell viability was measured using CCK8. Data represent the mean (± standard deviation, SD) of three independent experiments, each performed in triplicate. Error bars indicate SDs. Asterisks indicate the statistical significance of each concentration of N8-acetylspermidine versus distilled water (* P<0.05, ** P<0.01; Tukey’s test)

Since some of the elderly-type fecal metabolites have been reported as risk factors for colorectal cancer, we assessed the effects of these metabolites on the progression of colorectal cancer using the colon cancer cell line HCT116. N8-acetylspermidine and propionic acid increased EGF signaling genes expression, suggesting that they might directly promote cell proliferation of colorectal cancer cells. In addition, N8-acetylspermidine, TMA, and choline induced the expression of the inflammatory cytokines IL-8 and IL-22, which are also known to induce cancer cell growth and survival (Figure 4). In fact, N8-acetylspermidine, which strongly induced IL-8 and IL-22, promoted cancer cell proliferation and inhibited cancer cell death caused by the anticancer drug oxaliplatin (Figure 5).

## Discussion

In recent years, many intestinal metabolomic analyses based on disease-related dysbiosis have been reported, leading to the clarification that the gut microbiota is strongly involved in the onset and progression of many diseases. However, the intestinal metabolites associated with age-related changes in the microbiota in healthy subjects have not been identified. In this study, we measured fecal metabolites using CE-TOF-MS in elderly subjects with age-matched and age-mismatched microbiota based on their gut microbiota composition and revealed bacteria with abundances correlated with the amount of each metabolite. Notably, compounds such as TMA, choline (precursor of TMA), and N8-acetylspermidine, which are known risk factors for age-associated diseases, were enriched in the feces of the E-GM group.

TMA is produced by gut bacteria mainly from food-derived choline or carnitine and is further metabolized to TMAO by the flavin-containing monooxygenase 3 (FMO3) enzyme in the liver. Many studies have reported that TMA/TMAO plays an important role in complex diseases such as CVD, renal diseases, metabolic disorders, neurological diseases, and cancer,^9,10^ Rath *et al*. reported that the putative choline TMA-lyase *cutC* gene was widely distributed in human intestinal bacteria (in particular *Gamma-* and *Deltaproteobacteria, Clostridia*, and some *Bacilli*), and the putative carnitine oxygenase *CntA* gene was observed in *Gammaproteobacteria*, especially *E. coli* and *Klebsiella*.^11^ Jie *et al*.　performed fecal metagenomic analysis of 218 individuals with atherosclerotic cardiovascular disease (ACVD) and 187 healthy controls. *Enterobacteriaceae* (including *E. coli, Klebsiella* spp. and *Enterobacter aerogenes*) were characteristically enriched in ACVD patients with TMA-producing genes.^12^ In addition, the most abundant form of choline in the diet is phosphatidylcholine. It has been reported that many choline-utilizing gut microorganisms can hydrolyze phosphatidylcholine, further converting the released choline to TMA.^13^ These reports suggest that age-related increase such as *Proteobacteria* may induce elevated choline and TMA production in the E-GM group and subsequently trigger chronic inflammatory diseases such as CVD. Actually, we found a positive correlation between fecal TMA and some *Proteobacteria* such as *Klebsiella oxytoca* (*r* = 0.53, Q value = 0.069), *E. coli* (*r* = 0.38, Q value = 0.268) and *Escherichia sp*. (*r* = 0.355, Q value = 0.268).

In this study, primary bile acid (BA), cholic acid, was elevated in the A-GM group. Primary BAs are synthesized from cholesterol in the liver and further metabolized by the gut microbiota to secondary BAs via deconjugation, dehydrogenation, and dehydroxylation in the gut.^14^ It is well known that secondary BAs exert carcinogenic stress, such as DNA damage, in host cells. However, we could not find known secondary BA-producing bacteria in both group, indicating that secondary BAs might be at low level in these healthy subjects. Actually, we measured bile acid composition using same fecal samples and confirmed that the primary BAs (CA, CDCA) showed a high level in the A-GM group, but no difference was observed in the secondary BAs (DCA, LC, UDCA) (data not shown). Interestingly, it has been reported that elevated TMAO in the liver suppresses the expression of the BA biosynthesis gene *cholesterol 7α-hydroxylase* (*CYP7A1*).^15^ Chen *et al*. reported that resveratrol (RSV), a natural phytoalexin with antiatherosclerosis effects, attenuated TMAO-induced atherosclerosis in mice by decreasing TMAO levels and increasing hepatic BA biosynthesis via gut microbiota remodeling. RSV induced gut microbiota changes by increasing *Bifidobacterium* and *Lactobacillus* and suppressed TMA/TMAO production in the gut and liver. At the same time, decreased TMAO production by RSV treatment caused an increase in *CYP7A1* expression in the liver, promoting biosynthesis of BAs, resulting in fecal excretion of BAs and suppressing excessive cholesterol accumulation in the liver.^16^ Taken together, elevated fecal primary BAs in the A-GM group may reflect BA biosynthesis via decreased TMAO production. Inhibiting fecal TMA production with age may be effective in preventing not only arteriosclerosis but also metabolic disorders.

In contrast, risk factors for colorectal cancer, such as N8-acetylspermidine and TMA, were detected at high levels in the E-GM group. Polyamine metabolites (putrescine, spermidine, and spermine) are known to have many important roles in cell proliferation and differentiation.^17^ Elevated levels of polyamines have been associated with breast, colon, lung, prostate, and skin cancers.^18^ Each polyamine has undergone various modifications *in vivo*, and acetylation has been suggested to be associated with cancer progression.^19^ N8-acetylspermidine and N1-acetylspermidine have been reported to be enriched in the tumor region of colorectal cancer patients.^20^ In this study, there was no significant difference in N1-acetylspermidine, but the level of this metabolite tended to be higher in the E-GM group than in the A-GM group. Likewise, microbial enzyme activity related to TMA production from choline in feces was upregulated in colon cancer patients, suggesting a relationship between choline metabolism and colon cancer.^21^ It has further been suggested that endogenous TMA in the gut can be converted to more toxic metabolites that can drive abnormal cellular proliferation in humans.^10^ We observed that the elderly type metabolites induced the expression growth factors and cytokines IL-8 and IL-22, known as inducers of cancer cell growth.^22,23^ IL-22 has also been reported to induce resistance to anticancer drugs in colon cancer cells by increasing IL-8 expression.^24^ According to these reports, N8-acetylspermidine induced cancer cell growth and chemoresistance in colon cancer cells, suggesting that elevated N8-acetylspermidine has the potential to promote the progression of colorectal cancer.

Importantly, choline and TMA suppressed tight junction-related gene expression in normal colonic epithelial cells, indicating that these metabolites could penetrate the intestinal barrier and spread throughout the body via the bloodstream. Increased intestinal permeability dependent on aging is considered to be one of the critical factors for promoting chronic inflammation (inflammaging) and related diseases.^25^ These results suggest that age-related gut microbial changes in healthy subjects might be a trigger for the induction of age-associated systemic diseases through increased intestinal permeability.

This study suggests that age-related changes in the gut microbiota in healthy individuals, especially in the elderly, cause or exacerbate systemic age-related diseases through specific metabolites. At the same time, it has also been shown that chronic inflammation-related diseases could be prevented by suppressing age-related changes in gut microbiota composition in the elderly. For example, it has been clarified that the abundances of TMA-producing bacteria were decreased in herbivores compared with carnivores and omnivores.^26^　However, since there are some limitations in this study, we consider these result as preliminary and need further detailed analysis. One is the small sample size. In this study, we analyzed the gut microbiota profile of 453 healthy Japanese subjects and found only about 40 elderly subjects classified as age-mismatched gut microbiota composition clusters (cluster 3 and 4). In the next analysis, it will be necessary to confirm the results using more samples. At the same time, it is necessary to acquire the subject’s dietary habits, clinical characteristics, medicine information, etc., to show the objectivity of healthy subjects. Contrary to this study, it is also need to investigate the fecal metabolites of adult subjects with aged-gut microbiota composition. In the future, it will also be necessary to evaluate the relationship of the elderly-type metabolites identified in this study with inflammaging, CVD, and colon cancer progression using aged mice or various disease model mice.

## Materials and methods

### Gut microbiota analysis

The 16S rRNA gene sequencing data of 453 healthy Japanese subjects were obtained from DDBJ under accession numbers DRA004160 and DRA005774. The sequences were analyzed using the QIIME software package, version 1.9.0, as previously described.^3^ After assignment of each operational taxonomic unit (OTU) to bacterial species, PCoA based on Jensen–Shannon divergence (JSD) and subsequent clustering were performed using R, version 3.2.4.^27^

### Metabolomic analysis and fecal metabolite concentration determination

Metabolomic analysis was conducted using the Basic Scan package from Human Metabolome Technologies, Inc. (HMT; Yamagata, Japan) using CE-TOF-MS. Fecal metabolites were extracted　by vigorous shaking with nine volumes of sterilized water containing the internal standard. The mixture was centrifuged, and the upper aqueous layer was centrifugally filtered according to the manufacturer’s instructions. Peaks detected by CE-TOF-MS were extracted using automatic integration software (MasterHands, Keio University, Japan) to obtain peak information, including m/z, migration time (MT), and peak area. The peaks were annotated with putative metabolites from the HMT metabolite database based on their MTs in CE and m/z values determined by TOF-MS. The tolerance range for the peak annotation was configured at ± 0.5 min for MT and ± 10 ppm for m/z. The relative area of each peak was calculated and compared between the two groups based on Welch’s t-test. The concentration of each metabolite was calculated relative to the concentration of the internal standard. Each fecal metabolite concentration in three samples was measured using the standard substances by another CE-TOF-MS experiment, and the levels were calculated based on the relative peak area in the remaining 29 samples.

### Reagents

Choline chloride (C0329:Tokyo Chemical Industry), trimethylamine (92262:Sigma-Aldrich), N8-acetylspermidine dihydrochloride (A3658: Sigma-Aldrich), cholic acid (032–03042: FUJIFILM Wako), and propionic acid (169–04723: FUJIFILM Wako) were purchased from the companies indicated.

### Cell culture

Normal HCoEpiCs were purchased from ScienceCell Research Laboratories (Carlsbad, CA, USA). The HCoEpiCs were generated from human colonic tissues, cryopreserved at passage one, and delivered frozen. All experiments were performed within 10 passages after obtaining the cell line. The cells were maintained in Colonic Epithelial Cell Medium (CoEpiCM, ScienceCell Research Laboratories) in a humidified 5% CO_2_ atmosphere at 37°C. The human colorectal cancer-derived IEC line HCT116 was obtained from American Type Culture Collection (Rockville, MD). Cells were grown in RPMI 1640 medium (11875093:Thermo Fisher Scientific) with 100 IU/ml penicillin, 100 μg/ml streptomycin, and 10% heat-inactivated FBS (2916554:MP Biomedicals) in a humidified 5% CO_2_ atmosphere at 37°C.

### Quantitative PCR

Total RNA was extracted from cultured cells using TRIzol reagent (Life Technologies), and reverse transcription and quantitative PCR were performed as previously described.^28^ Primers were used as follows: human GAPDH, 5ʹ-CAACTACATGGTTTACATGTTC-3ʹ (forward) and 5ʹ-GCCAGTGGACTCCACGAC-3ʹ (reverse); human claudin-4, 5ʹ-GCCGGCCTTATGGTGATAGTG-3ʹ (forward) and 5ʹ-CACCAGCGGATTGTAGAAGTCTTG-3ʹ (reverse); human occldin, 5ʹ-AAGAGTTGACAGTCCCATGGCATA-3ʹ (forward) and 5ʹ-AGGCTGCCTGAAGTCATCCAC-3ʹ (reverse); human IL-8, 5ʹ-AAGGAAAACTGGGTGCAGAG-3ʹ (forward) and 5ʹ-ATTGCATCTGGCAACCCTAC-3ʹ (reverse); human IL-22, 5ʹ-TGGCAAAGAAGGGCTGTCAG-3ʹ (forward) and 5ʹ-GCGGTGACCCTGGCATAGT-3ʹ (reverse); human EGF, 5ʹ- GTGCAGCTTCAGGACCACAA-3ʹ (forward) and 5ʹ-AAATGCATGTGTCGAATATCTTGAG-3ʹ (reverse); human VEGF, 5ʹ-GGCCAGCACATAGGAGAGATG-3ʹ (forward) and 5ʹ-AGGCCCACAGGGATTTTCTT-3ʹ (reverse); human bFGF, 5ʹ-CGACCCTCACATCAAGCTACA-3ʹ (forward) and 5ʹ-AACGGTTAGCACACACTCCTT-3ʹ (reverse); human EGFR, 5ʹ-TGACTGAGGACAGCATAGACGA-3ʹ (forward) and 5ʹ-GGGCTGGACAGTGTTGAGATAC-3ʹ (reverse); human FGFR, 5ʹ-GGCTGCCAAGACAGTGAAGTT-3ʹ (forward) and 5ʹ-GGTTGATGCTGCCGTACTCAT-3ʹ (reverse).

### Cell proliferation assay

Cell proliferation assays were carried out using Cell Counting Kit 8 (CCK8) (Dojindo). HCT116 cells were plated in 96-well plates at a density of 2 × 10^3^ cells per well and cultured in serum-free medium. Twenty-four hours later, the cells were treated with the indicated concentration of N8-acetylspermidine. At the indicated time points, cell numbers were measured using CCK8 according to the manufacturer’s instructions.　For cell proliferation immunostaining, HCT116 cells were fixed with 4% paraformaldehyde in PBS, permeabilized by 0.1% Triton X-100, treated with 2%BSA in PBS for 10 min at room temperature, respectively. Then, cells were incubated with Ki-67 antibody (eBioscience, 14–5698-82) and fluorescence-conjugated secondary antibody (Thermo Fisher Scientific, A-11006), and observed under fluorescence microscope.

### Cell viability assay

The chemotherapeutic drug oxaliplatin (Sigma-Aldrich, St Louis, MO, USA) was used in this assay. HCT116 cells were seeded on 96-well plates (1 × 10^3^ cells per well) in growth medium. Twenty-four hours later, the cells were treated with 20 μM oxaliplatin and the indicated concentration of N8-acetylspermidine and cultured for an additional 48 h. Then, cell numbers were measured using CCK8 according to the manufacturer’s instructions.

### Statistical analysis

Intergroup differences in bacterial species between the adult- and elderly-type microbiota groups were analyzed by the linear discriminant analysis (LDA) effect size (LEfSe) method^29^ with default settings on the website (https://huttenhower.sph.harvard.edu/galaxy/root).

Values of *p* < 0.05 were considered statistically significant.

Spearman correlation coefficients between each metabolite and gut bacterial species were calculated using IBM SPSS Statistics, version 22.0, statistical software package (IBM Corp., Armonk, NY, USA). Multiple testing correction, modified Benjamini–Hochberg (BH) procedure,^30^ was performed using the R package ‘qvalue’ (http://github.com/jdstorey/qvalue) on R software version 3.6.0. Q value <0.1 was considered statistically significant.

## Supplementary Material

Supplemental Material
