# Supplementary material for: Enriched metabolites that potentially promote age-associated diseases in subjects with an elderly-type gut microbiota
Source: Gut Microbes. 2021 Jan 11;13(1):1865705. doi: 10.1080/19490976.2020.1865705 (PMC7808425; doi:10.1080/19490976.2020.1865705)
Supplement: Supplemental Material [file KGMI_A_1865705_SM6091.zip › supplement/Sup_Fig_Revised.pdf]

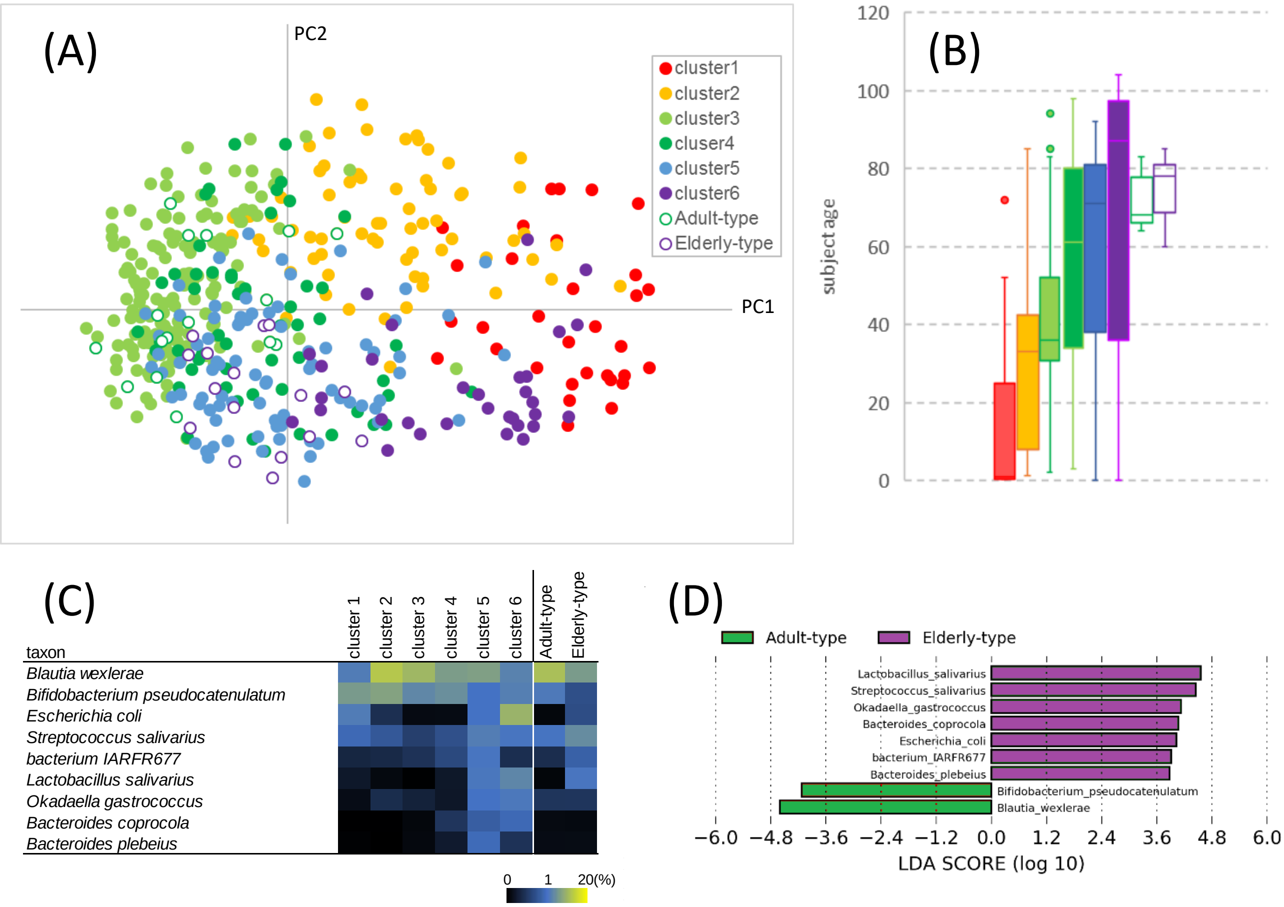

Figure 1.
Fecal microbiota composition

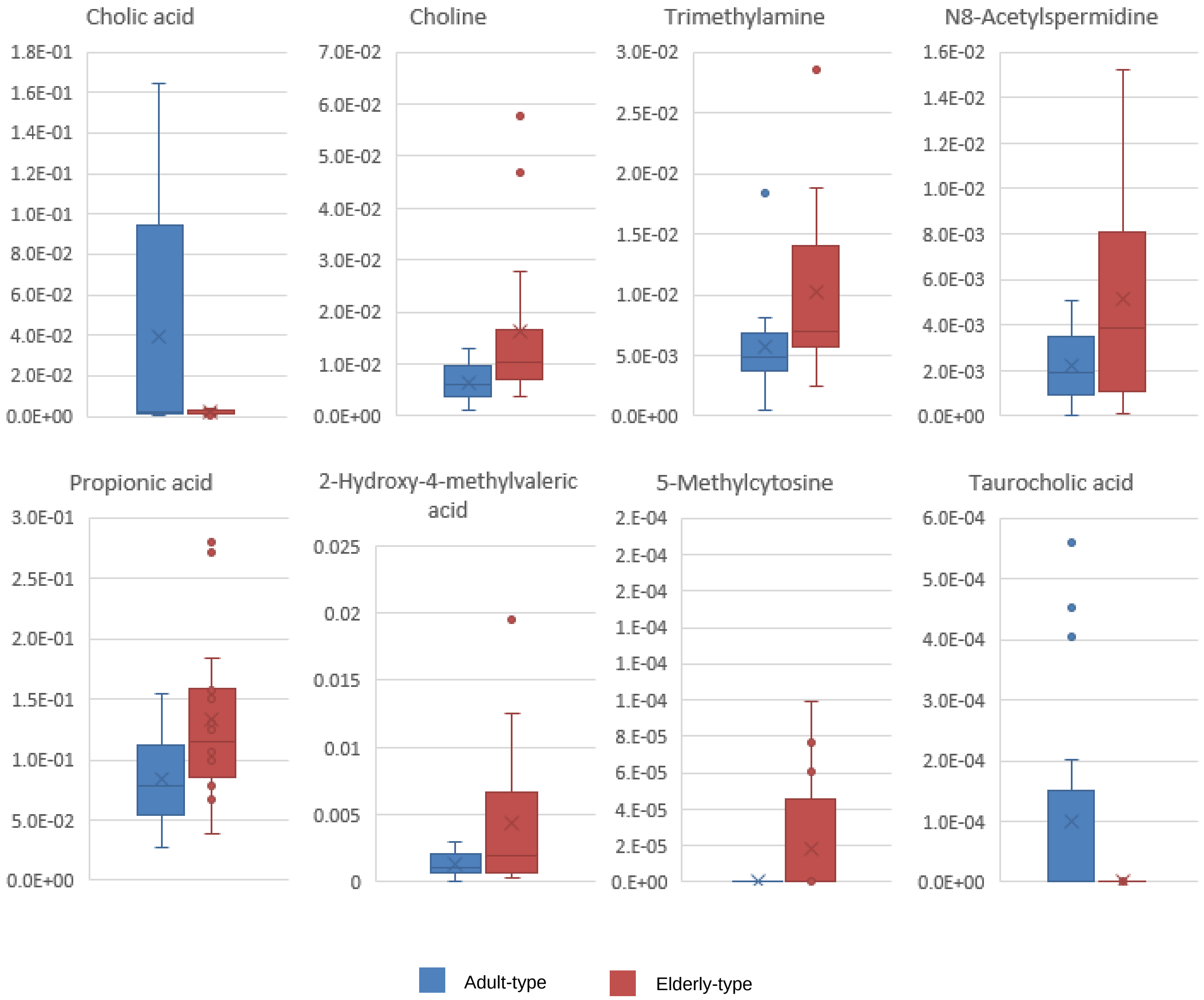

Figure 2.
Eight fecal metabolites were significantly different between the adult- and elderly-type gut microbiota

Relative mRNA expression

		Metabolite (μM)	claudin1	claudin4	occludin	TJP1	Muc1
	Control	-	1.0	1.0	1.0	1.0	1.0
Elderly type	TMA	1	1.2	1.0	0.9	1.0	1.1
		10	* 0.9	** 0.7	** 0.7	** 0.7	1.0
		100	1.0	** 0.8	** 0.7	1.1	* 0.9
	Choline	0.5	* 0.9	** 0.6	** 0.6	0.8	** 0.7
		10	0.9	** 0.7	** 0.6	* 1.8	* 0.8
		50	1.0	** 0.8	** 0.7	1.1	* 0.9
	N8-Acetylspermidine	2	1.0	1.0	1.1	1.1	0.9
		5	1.1	1.0	1.0	** 2.4	1.1
		10	* 1.2	1.0	1.0	** 1.9	1.2
	Propionic acid	10	1.0	** 0.5	** 0.6	* 0.8	1.2
		100	1.0	0.9	1.0	1.2	1.0
		1000	1.0	1.1	1.3	* 1.5	0.9
Adult type	Cholic acid	0.5	** 0.6	1.2	1.2	* 1.2	** 1.9
		5	** 0.7	* 1.6	1.4	* 1.2	** 1.8
		25	** 0.7	** 1.9	** 1.8	1.2	* 1.6

Color scale: 0.5 / 1.0 / 2.0

Figure 3.
Downregulation of epithelial barrier-related gene expression by elderly-type gut metabolites in normal colonic epithelial cells.

Relative mRNA expression

		Metabolite(uM)	IL-8	IL-22	EGF	VEGF	bFGF	EGFR	FGFR
	Control	-	1.0	1.0	1.0	0.9	1.0	0.9	1.0
Elderly type	TMA	1	** 3.3	1.4	* 0.3	** 0.5	** 0.3	** 0.4	** 0.3
		5	** 3.8	1.5	1.1	** 1.6	1.0	** 1.4	1.3
		10	** 3.3	1.5	1.4	1.2	1.2	1.2	1.2
	Choline	1	** 1.9	0.8	1.1	* 0.7	** 0.6	** 0.5	** 0.6
		5	** 2.1	** 5.1	* 0.4	** 0.5	** 0.5	** 0.4	** 0.4
		20	** 2.7	** 11.9	0.8	0.7	0.9	1.0	0.8
	N8-Acetylspermidine	2	0.5	1.2	1.3	** 1.5	1.3	* 1.3	* 1.3
		5	** 4.1	1.2	1.4	1.3	1.1	1.1	1.2
		10	** 5.1	** 22.5	* 1.7	0.8	* 0.8	** 0.6	* 0.7
	Propionic acid	10	1.0	0.7	* 1.5	0.8	** 1.3	1.1	1.6
		100	1.0	1.0	** 3.4	* 0.7	* 1.1	* 0.7	* 2.0
		1000	1.2	1.4	1.4	** 0.5	** 1.9	1.1	** 3.2
Adult type	Cholic acid	5	1.3	* 1.3	1.4	** 1.6	* 1.3	1.1	1.1
		20	1.0	** 0.5	1.3	1.0	1.0	* 1.4	0.8

down
1.0
up

Figure 4.
Induction of cytokines and growth factors expression by the elderly-type gut metabolites in colon cancer cells.

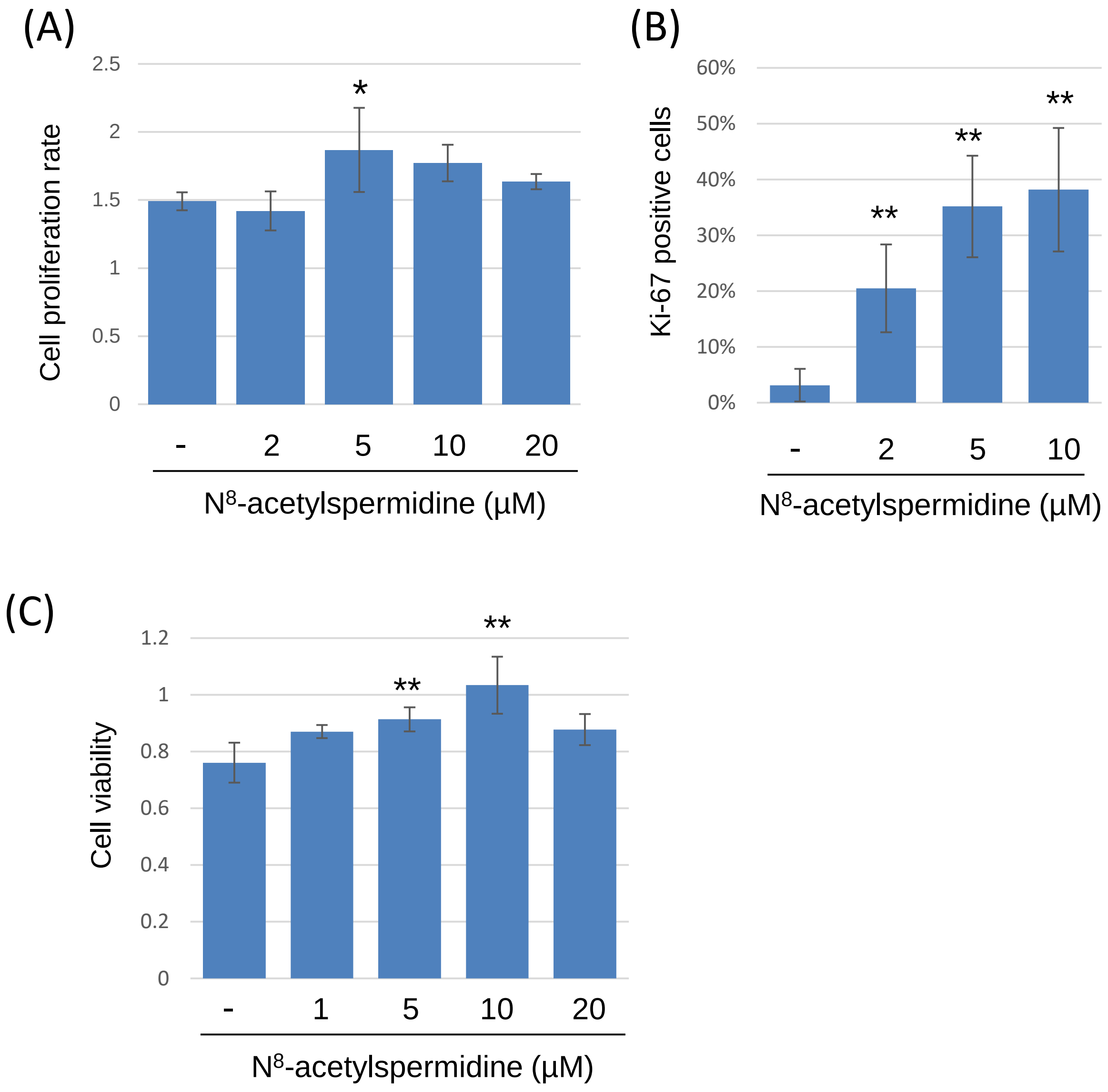

Figure 5.
Induction of cell proliferation (A,B) and chemoresistance (C) to colon cancer cells by N8-acetylspermidine.
